# Assessing incarcerated women’s physical and mental health status and needs in a Swiss prison: a cross-sectional study

**DOI:** 10.1186/s40352-022-00171-z

**Published:** 2022-02-22

**Authors:** Aurélie Augsburger, Céline Neri, Patrick Bodenmann, Bruno Gravier, Véronique Jaquier, Carole Clair

**Affiliations:** 1 Centre for Primary Care and Public Health (Unisanté), Department of Training, Research and Innovation, University of Lausanne, Lausanne, Switzerland; 2 Service of Correctional Medicine and Psychiatry, University Hospital of Lausanne CHUV, Lausanne, Switzerland; 3 Centre for Primary Care and Public Health (Unisanté), Department of Vulnerabilities and Social Medicine, University of Lausanne, Lausanne, Switzerland; 4 Centre for Criminological Research (CRRC), University of Neuchâtel, & School of Health Sciences Fribourg (HEdS-FR), HES-SO University of Applied Sciences and Arts Western Switzerland, Neuchâtel/Fribourg, Switzerland

**Keywords:** Incarcerated women, Prison, Women’s health, Disparities, Gender

## Abstract

**Background:**

Women make up 5% of the European prison population on average. Almost invisible in prison and health research, and suffering the stigma associated with female offending, incarcerated women are often forgotten, and their specific healthcare needs remain much ignored. Combining face-to-face survey interviews and medical chart data, we aim to assess the health status, healthcare needs, and access to preventive medicine of women incarcerated in Switzerland.

**Results:**

Sixty incarcerated adult women participated in a cross-sectional study to assess their life and incarceration histories, physical and mental health problems, medication, and use of medical services. Eligibility criteria were (a) an incarceration of at least four weeks and (b) the ability to provide written informed consent. Exclusion criteria were psychiatric instability and insufficient language competence.

Women’s average age was 34.3 years old (*SD* = 9.8); 45.0% of them were born in Switzerland, 33.3% in Europe and 15.0% on the African continent. Overall, 61.7% of women self-reported physical or mental health problems and 13.3% indicated they were once diagnosed with a sexually transmitted infection. Further, 78.3% of women were active cigarette smokers; more than one in three women reported alcohol use problems and almost one in two women had used at least one illicit drug in the year before incarceration. Depression and perceived stress scores were above clinical cut-off points for more than half of interviewed women. When asked how they rated their health, 68.3% of women felt it had worsened since incarceration. All but four women had accessed prison medical services; however, our study does not indicate whether women’s use of healthcare was indeed adequate to their needs.

**Conclusions:**

This study demonstrated incarcerated women’s poor health and health-risk behaviours. Structural changes and gender-responsive health promotion interventions have the potential to improve the health of incarcerated women and help them return to the community in better health.

## Background

Prison health, for long a secondary issue for prison institutions, is now recognized as a public health issue (Ramaswamy & Freudenberg, [52]). With the social disadvantage and serious health problems of the prison population, access to quality health services in prison is of vital importance (Ismail, Lazaris, O’Moore, Plugge, & Stürup-Toft, [29]). Yet prison systems are required to meet the health needs of individuals in prison with limited resources and while facing important organizational and ethical challenges (Elger, [16]). Incarcerated women have more, and more specific health problems than men, which places additional constraints on prison health services (van den Bergh, Gatherer, Fraser, & Moller, [61]). In Switzerland, as elsewhere in Europe (Walmsley, [63]) one in 20 incarcerated persons is a woman. Outnumbered by men in criminal justice and correctional populations, the specificities of women’s trajectories are often ignored (Jaquier & Vuille, [31]), and their specific health and social needs remain much neglected (Braithwaite, Treadwell, & Arriola, [7]; Swavola, Riley, & Subramanian, [59]).

Research has highlighted the complex and chronic health problems faced by incarcerated women before, during, and after incarceration (Braithwaite, Arriola, & Newkirk, [8]; Jaquier, Neri, Augsburger, & Clair, [30]; WHO & UNODC, [65]). Chronic conditions (e.g., asthma, cancers, cardiovascular diseases) have been found more prevalent among incarcerated women than among incarcerated men (Dean, [14]; McQueen, [42]). Gender differences persist even when accounting for demographic and socioeconomic factors and substance use (Binswanger et al., [5]). Incarcerated women display a large array of mental health problems, from depression to posttraumatic stress, often the consequences of violence and abuse (van den Bergh, Plugge, & Yordi Aguirre, [60]). The uniqueness of women’s trauma histories plays a critical role in explaining gender differences in mental health in prison, but also in substance abuse (Grella, Lovinger, & Warda, [22]; Moloney, van den Bergh, & Moller, [44]). Indeed, women entering prison have strikingly high rates of substance use problems (Binswanger et al., [5]). Also smoking is highly prevalent in this population thus putting incarcerated women at higher risk of smoking-related diseases (Cropsey, Eldridge, & Ladner, [12]; Plugge, Douglas, & Fitzpatrick, [50]). Last, because of sexual risk behaviours, drug use, sexual abuse, and often marginalized and socially deprived backgrounds, incarcerated women are at increased risks for sexual and reproductive health diseases, including cancers and sexually transmitted infections (STIs) (De Groot & Maddow, [13]; Macalino, [39]).

Incarcerated women’s health problems are further compounded by their limited access to healthcare prior to incarceration (Conklin, Lincoln, & Tuthill, [11]). Women’s physical and mental health is often poor upon prison admission and susceptible to deteriorate over time due to both the prison physical environment and the subjective experience of incarceration (Harner & Riley, [24]). Extant research has underscored that incarcerated women have a low utilization of healthcare services in the community and that, while in prison, they seek medical services to a greater extent than men (Lindquist & Lindquist, [38]; Staton, Leukefeld, & Webster, [58]). For many women, detention creates access to adequate healthcare services, possibly for the first time (Anderson, [1]). Research has therefore also underscored the essential role of screening and preventive medicine interventions during incarceration (WHO & UNODC, [65]).

Women’s increased vulnerability in prison poses unique challenges to medical professionals, when the incarceration exacerbates women’s mental health problems and traumatic experiences. Imprisoned women often serve a short sentence, resulting in a high turnover rate and thus complicating planning for continuous care. All along medical services for incarcerated women remain limited and often not tailored to their specific needs (Lewis, [37]; van den Bergh, Gatherer, & Møller, [62]).

That being said, most of the research on incarcerated women’s health has been conducted in North America or the United Kingdom and very limited research is available elsewhere in Europe (MacDonald, [40]). Yet women’s criminal trajectories, biographical and individual characteristics vary across countries, as do prison systems and accessibility of healthcare services. As such, extant international findings do not necessarily apply from one country to another.

So far, little is known about the current health status of women incarcerated in Switzerland. A few studies have compared the mental and physical health of men and (a few) women on remand (Eytan et al., [18]; Moschetti et al., [45]; Wolff et al., [66]), others have focused solely on women under forensic psychiatric care (Krammer, Eisenbarth, Fallegger, Liebrenz, & Klecha, [33]; Krammer, Linder, Peper, Covington, & Klecha, [34]; Rossegger et al., [53]), and a single study has described the health status of elderly (50+ years old) women in prison (Handtke, Bretschneider, Elger, & Wangmo, [23]). Specifically examining the case files of incarcerated women under forensic-psychiatric care, three studies conducted in the German-speaking part of Switzerland identified multiple aversive and potentially traumatic events in women’s childhood and adulthood and a history of psychiatric treatment, although two of these three studies comprised ≤20 women (Krammer et al., [33]; Krammer et al., [34]; Rossegger et al., [53]). In the French-speaking part of Switzerland, three studies analysing medical records found more frequent mental health problems in women, while the comparisons of the rates of physical health and substance use problems between men and women yielded inconsistent results (Eytan et al., [18]; Moschetti et al., [45]; Wolff et al., [66]). Yet, no study that we know of has collected comprehensive health data on incarcerated Swiss women’s health problems and needs, and overall prison experience using face-to-face survey interviews. There is a dearth of research to inform prison health and public health initiatives.

In 2017, 6907 persons were incarcerated in Swiss prisons, of which 382 were women (5.6%) (Office fédéral de la statistique, [48]). Switzerland holds 92 penal institutions, with a capacity ranging from five to 398 individuals. Two prisons are specifically dedicated to women, the *Hindelbank* prison located in the German-speaking part of Switzerland and the *La Tuilière* prison – where the study took place – located in the French-speaking part of Switzerland. Most women are housed in one of these two prisons, although some might be incarcerated in small sections of men’s prisons, which leads to gender discriminations in many areas including healthcare (European Committee for the Prevention of Torture and Inhuman or Degrading Treatment or Punishment, [17]; Penal Reform International & Association for the Prevention of Torture, [49]). Access to quality healthcare vary greatly across prisons: small prisons are not staffed with healthcare professionals every day, the number of trained professionals remains too low, overcrowding is a perpetual issue as is the increased use of short prison sentences, and interactions between judicial or correctional authorities and medical professionals have proven complicated (Chatterjee, Wolff, Baggio, & Gétaz, [9]).

Policy documents at different levels have underlined the complex health and social problems faced by incarcerated women in Switzerland and recognized the need to attend to their specific needs. Yet so far little has been done to move beyond drafting recommendations. As an attempt to address critical gaps in research and correctional clinical practice, we designed an observational study assessing women’s health status, healthcare needs, and access to preventive medicine. Exploring incarcerated women’s complex health problems and how these are further negatively impacted by their prison experience is the first step towards designing initiatives that meet the specific needs of this particularly vulnerable prison population, therefore contributing to reducing prison health and public health gender disparities.

## Methods

### Research site

Sixty incarcerated women participated in a cross-sectional study combining survey data collected face-to-face and medical record data to assess their health status and needs. They were recruited from the prison *La Tuilière*. The prison is located in the small town of Lonay, in the French part of Switzerland, and offers 61 beds to women in pre-trial detention or serving a prison sentence, alongside a small section for men in pre-trial detention. The prison medical unit of *La Tuilière* is located in a dedicated wing of the prison and is open daily providing ambulatory care to incarcerated women and men; the medical staff comprises 4.9 full-time equivalent nurses, two psychiatrists on site during working days, and one part-time psychologist. Medical professionals in the state where the study took place are independent from both the prison administration and the criminal justice system. However, this setting is rare in Switzerland where most prison medical units depend on judicial or correctional authorities (Chatterjee et al., [9]).

Women are systematically seen by a nurse within 48 h of their incarceration, and by a general practitioner (GP) within 7 to 21 days. These entry examinations allow health professionals to screen for somatic and infectious diseases and conduct a preliminary evaluation of a woman’s mental health. By appointment, a GP comes in two to three times a week for a half-day; similarly, physical therapists are available once a week for consultation and a dentist once a month. Gynaecologists, podiatrists, midwives, and child psychiatrists are available upon request. For other specialty consultations, a transfer to the local university hospital is necessary. Whenever incarcerated women want a medical consultation, they are to send a written request to the prison medical unit.

### Data collection

Recruitment flyers advertising for a study on incarcerated women’s health were posted in female housing units and various locations throughout the prison medical unit between February and November 2017. Additionally, two female research associates (AA and CN) gave oral presentations about the study to incarcerated women at their workplaces inside the prison. To ensure adequate participation, the research associates also approached women individually or in small groups to present the study. Interested women then met with one of the research associates, within the prison medical unit, to discuss the specifics of study participation and determine eligibility. To be eligible, a woman had to be (a) 18 years or older, (b) incarcerated for at least four weeks, and (c) able to provide written informed consent. Exclusion criteria were psychiatric instability as established by the correctional health team and insufficient language competence.

Eligible women who provided informed consent were invited to take part in a research survey, administered face-to-face using the secure web application REDCap® (Harris et al., [25]). The research interviews were conducted by one of two female research associates, a study nurse (CN) and a medical resident (AA). Both had professional experience working in prison settings and received extensive training in research ethics, interviewing incarcerated persons, and building an alliance with research participants. The research associates, however, were not working as healthcare providers any more at the time of the study. Interviews were conducted in French or English. Unfortunately, we were not able to include women participants who were not fluent in either of these two languages mostly due to lack of funding for interpreters but also to the length and complexity of the questionnaire. All interviews took place in a private room within the prison medical unit in the absence of any security staff and lasted approximately 1.5 h. At the end of their interview, women participants were asked to provide access to their prison medical record to complement their health profile. Physical measurements (i.e., height, weight, blood pressure) were also taken. Women participants did not receive financial compensation for their time, but snacks were available during the interviews.

Survey data and physical measurements were entered directly in the secure web application (REDCap®). Women were identified by a unique code number to maintain medical confidentiality. All data extracted from the medical charts were also entered directly into REDCap® using women’s individual code number. The Research Ethics Committee of the principal investigators’ institutions approved all study procedures.

### Survey data

The study survey was modelled after the New South Wales Inmate Health Survey (Indig et al., [28]) designed to examine social determinants of health (e.g., age, country of birth, motherhood, income) and prison experience (e.g., length of prison stay, prior incarceration), self-reported physical and mental health problems including STIs, health-risk behaviours (e.g., smoking, alcohol and drug use in prison, low physical activity, poor diet), healthcare use (e.g., consultations with various medical professionals), and preventive health measures (e.g., PAP smear, chest X-ray). We also included a series of validated clinical assessments to screen women for mental health problems and substance use; these assessments are described below.

#### Anxiety

The Generalized Anxiety Disorder Screener (GAD-7) (Spitzer, Kroenke, Williams, & Löwe, [57]) is a brief measure consisting of 7 items used to screen for and assess the severity of generalized anxiety disorder over a period of two weeks. Women participants indicated how often they were bothered by each item, from 0 = *not at all* to 3 = *nearly every day*. A severity score was calculated by summing all items, with higher scores indicating greater anxiety severity. Cronbach’s *α* = 0.88 in this study. Scores of 5, 10, and 15 were taken as the cut-off points for mild, moderate, and severe levels of anxiety, respectively.

#### Depression

The Patient Health Questionnaire 9 (PHQ-9) (Kroenke, Spitzer, & Williams, [35]), frequently used in primary care, assesses each of the nine DSM-IV criteria for depression over a period of two weeks. Women participants indicated how often they were bothered by each item, from 0 = *not at all* to 3 = *nearly every day*. A severity score was calculated by summing all items, with higher scores indicating greater depression severity. Cronbach’s *α* = 0.83 in this study. Scores of 5, 10, 15 and 20 were taken as the cut-off points for mild, moderate, moderately severe, and severe levels of depression, respectively. A diagnosis of major depression is considered if five or more of the nine items are endorsed for at least “more than half the days,” and provided that one of these items is anhedonia (item 1) or depressed mood (item 2). A diagnosis of other depression is considered if two, three or four of the nine items are endorsed for at least “more than half the days,” and provided that one of these items is anhedonia (item 1) or depressed mood (item 2). For both variables, suicidal thoughts (item 9) counts if endorsed, regardless of frequency.

#### Perceived stress

The Perceived Stress Scale (PSS-10) (Cohen & Williamson, [10]; Dupret & Bocéréan, [15]) assesses the frequency with which life situations are generally perceived as “threatening, unpredictable, uncontrollable and painful,” but it does not assess stress symptoms or stress reactions to specific events. The 10 items simply ask about participants’ feelings and thoughts over the last month and events are rated from 0 = *never* to 4 = *very often*. A total score was calculated by summing all items, with higher scores indicating greater perceived stress. Cronbach’s *α* = 0.81 in this study. Scores between 0 and 20 were considered indicative of low perceived stress, scores between 21 and 26 indicative of moderate perceived stress, and scores greater than 27 indicative of high perceived stress.

#### Smoking behaviours

Smoking status was self-reported; women who reported being current smokers and had smoked ≥100 cigarettes in their lifetime were considered active smokers. The Fagerström Test for Nicotine Dependence (FTND) (Heatherton, Kozlowski, Frecker, & Fagerstrom, [26]) was administered to measure the intensity of physical addiction to nicotine. The total score was created by summing all six item scores, Cronbach’s *α* = 0.65 in this study. The FTND categorical variable allocates participants into four groups according to their total score, namely “low dependence” (scores 1–2), “low to moderate dependence” (3–4), “moderate dependence” (5–7), and “high dependence” (≥8).

#### Alcohol use problems

The severity of alcohol use problems for the 12-month period prior to incarceration was assessed using the Alcohol Use Disorders Identification Test (AUDIT) (Babor, Higgins-Biddle, Saunders, & Monteiro, [3]). The total score was created by summing all 10 items, with higher total scores indicative of greater alcohol use problems, Cronbach’s *α* = 0.87 in this study. We allocated participants who drank into four levels of risk for alcohol use problems based on their total score: namely “low risk drinking” (scores 1–7), “alcohol use in excess of low risk guidelines” (8–15), “harmful and hazardous drinking” (16–19), and “possible alcohol dependence” (20–40).

#### Drug use problems

The severity of drug use problems for the 12-month period prior to incarceration was assessed using the 10-item Drug Abuse Screening Test (DAST-10) (Skinner, [56]). The sum score of all 10 yes/no items was calculated by summing affirmative answers, with higher scores indicative of greater drug use problems. Cronbach’s *α* = 0.81 in this study. The DAST-10 allocates participants who used drugs into four levels of risk for drug use problems based on their total score, namely “low risk” (scores 1–2), “moderate risk” (3–5), “substantial risk” (6–8), and “severe risk” (9–10).

### Physical measurements

#### Body mass index (BMI)

Body weight and height at the time of the survey were measured with participants standing without their shoes in light indoor clothes. BMI was calculated by dividing the body weight by the square of the body height (kg/m^2^). Based on the World Health Organization (WHO) criteria (WHO, [64]), underweight was identified as BMI < 18.5 kg/m^2^, overweight as BMI 25.0–29.9 kg/m^2^, and obese as BMI ≥ 30.0 kg/m^2^.

#### Blood pressure

Blood pressure at the time of the survey was measured on the right arm with an appropriately sized cuff and by one of the research associates, both trained medical professionals. Both diastolic and systolic blood pressure were recorded to the nearest 2 mmHg.

### Health-related data extracted from medical charts

To complete their health profile, women participants provided access to their prison medical records. Data were extracted from paper medical charts and coded to guarantee women’s confidentiality. In this paper, we examined data on BMI and blood pressure at incarceration to allow comparisons with values obtained at the time of the survey. We looked at prescribed medications and, last, at women’s healthcare visits to discuss women’s access to healthcare prior to and during incarceration.

### Participants

Between February and November 2017, 136 women were incarcerated at *La Tuilière*. The research associates reviewed the files of 130 incarcerated women for possible inclusion; however, 40 women left before they could be contacted. Of the remaining 90 women, eight women were not eligible because they had been incarcerated for less than four weeks and three could not be included in the study due to medical reasons. Nineteen women refused to participate, mainly because they were about to leave the prison or due to a lack of interest for the study.

Sixty women provided informed consent and took part in the study; one woman did not complete the survey (incomplete data) and it appeared later that one participant had been incarcerated for less than 4 weeks at the time of inclusion (protocol deviation). Medical chart data could be obtained for all women who answered the survey. The participation rate for this study was 44.0%. The size of this convenience sample was pre-specified in the protocol based on women’s turnover in prison and study resources.

### Analyses

Descriptive statistics were computed for demographic characteristics. Clinical assessment data were coded according to their respective guidelines and relevant cut-off points. Percentages, means (*M*) and standard deviations (*SDs*) or medians (*Mdn*) with interquartile ranges (IQRs) were calculated for each variable. Statistical analyses were performed using Stata Statistical Software (StataCorp 2015, Stata Statistical Software: Release 14.0, College Station, TX, StataCorp LLC).

## Results

### Incarcerated women’s socio-demographics, custodial regime and prison history

Socio-demographic characteristics, custodial regime, and prison history are shown in Table 1. The mean age of women participants was 34.3 years (*SD* = 9.8, range 21–64 years); 45.0% of them were born in Switzerland, 33.3% in Europe and 15.0% on the African continent.

**Table 1:** *Incarcerated Women’s Sociodemographics, Custodial Regime and Prison History (n = 60)*

Variables	Study sample	
	*n* or else specified	% or else specified
Mean age (*M*, *SD*)	34.3	9.8
Country of birth		
Switzerland	27	45.0
Europe	20	33.3
African continent	9	15.0
Other countries	2	3.3
Nonresponse	2	3.3
Marital status		
Single	31	51.7
Married	7	11.7
Separated/divorced	17	28.3
Widowed	5	8.3
Motherhood		
Any children	38	63.3
Children under 18	31	51.7
Mothers’ mean age (*M*, *SD*)	36.7	10.7
Highest educational qualification		
Less than mandatory education	16	26.7
Mandatory education	17	28.3
Apprenticeship	17	28.3
High school degree	5	8.3
University degree	5	8.3
Employment status, last 6 months prior to incarceration		
Not working	41	68.3
Working, part-time (< 5 days a week)	7	11.7
Working, full-time	12	20.0
Monthly household income, time of incarceration ^a^		
0–3000 CHF	42	70.0
3001–6000 CHF	9	15.0
6001–8000 CHF	3	5.0
> 8000 CHF	2	3.3
Does not know	3	5.0
Nonresponse	1	1.7
Current custodial regime		
Pre-trial detention	24	40.0
Anticipatory execution of prison sentence	7	11.7
Serving short prison sentence (< 12 months)	13	21.7
Serving prison sentence	16	26.7
Working in prison (% yes)	53	88.3
Prison trajectory		
Median length for prison stays at time of survey (in days; *Mdn*, *IQR*)	65.0	27.8
First prison stay (% yes)	34	56.7
Mean number of previous prison stays(*M*, *SD*; range: 1–15)	2.1	2.2
Incarcerated as a minor (% yes)	10	16.7

^a^10 CHF (Swiss franc) = 9.95 US dollars

Almost two in three women were mothers (63.3%), with most of them having children under 18 years old (81.6% of mothers, i.e. 51.7% of women participants). Educational qualifications among this population were low; 26.7% of women did not complete mandatory education and 28.3% did not pursue education beyond mandatory school years. Only 19 women were working part-time or full-time at the time of their incarceration, most of the participants were unemployed (68.3%).

For half the women (56.7%), their incarceration at *La Tuilière* was their first prison stay (mean number of prison stays = 2.1, range 1–15) and 10 women reported at least one prior incarceration as a minor. Most participants (40.0%) were held in pre-trial detention and the median length for prison stay at the time of the survey was 65 days (IQR = 27.8).

### Physical and mental health status

Close to one in three women rated her health as poor (20.0%) or very poor (11.7%) and 68.3% felt that incarceration had worsened their health (Table 2). When asked directly, 61.7% of women declared they suffered from a health problem. Almost half of the women (48.3%) self-reported a physical health problem; the most frequent self-identified diagnoses were rheumatic diseases (15.0%) and metabolic syndromes (13.3%; data not presented in tabular form). Eight women (13.3%) stated they were diagnosed with an STI at least once (e.g., HIV or hepatitis B or C infection). And 43.3% of the women self-reported mental health problems; the most frequent self-identified diagnoses were depressive (20.0%) and anxiety (20.0%) disorders (data not presented in tabular form). Most importantly, comorbidity was particularly frequent, with 35.0% of women reporting both physical health (including STIs) and mental health problems. Sixty-five percent of incarcerated women reported actual pain on the day of the survey, with an average pain intensity of 60.2 (*SD* = 16.7) on a 0–100 scale. At the time of incarceration, one in three women had a high BMI, precisely 15.0% of women were found to be overweight and 15.0% obese. We found an increase of the percentage of overweight women when comparing these initial BMI values to those calculated at the time of the survey (15.0% to 25.0%) and a corresponding decrease of the percentage of women in the normal weight range (46.7% to 41.7%).

**Table 2:** *Incarcerated Women’s Health Status and Problems According to Survey and Medical Charts Data (n = 60)*

Variables	Study sample	
	*n* or else specified	% or else specified
Self-reported health status		
Very good	9	15.0
Good	14	23.3
Fairly good	18	30.0
Poor	12	20.0
Very poor	7	11.7
Felt actual pain on the day of the survey (% yes)	39	65.0
Felt that incarceration worsened health (% yes) ^a^	41	68.3
Self-reported any health problems (% yes)	37	61.7
Physical health problems, STIs excluded (% yes)	29	48.3
STIs (% yes)	8	13.3
Mental health problems (% yes)	26	43.3
Physical and mental health problems (% yes)	21	35.0
BMI (kg/m2)		
*At interview*		
Mean BMI (*M*, *SD*)	24.9	5.7
BMI category		
Underweight	7	11.7
Normal Weight	25	41.7
Overweight	15	25.0
Obesity	9	15.0
No data	4	6.7
*At incarceration*		
Mean BMI (*M*, *SD*)	24.1	5.7
BMI category		
Underweight	7	11.7
Normal Weight	28	46.7
Overweight	9	15.0
Obesity	9	15.0
No data	7	11.7
Blood pressure (mmHg)		
*At interview* ^b^		
Mean systolic (*M*, *SD*; range: 98–150)	118.0	12.5
Mean diastolic (*M*, *SD*; range: 50–98)	76.7	9.9
*At incarceration* ^a^		
Mean systolic (*M*, *SD*; range: 90–157)	117.4	14.4
Mean diastolic (*M*, *SD*; range: 60–113)	78.2	9.6
Anxiety severity (GAD-7)		
Minimal or not anxious	15	25.0
Mild	14	23.3
Moderate	12	20.0
Moderately severe	18	30.0
Nonresponse	1	1.7
Depression severity (PHQ-9)		
Minimal or not depressed	8	13.3
Mild	13	21.7
Moderate	12	20.0
Moderately severe	14	23.3
Severe	12	20.0
Nonresponse	1	1.7
Perceived stress severity (PSS-10)		
Low	14	23.3
Moderate	35	58.3
High	10	16.7
Nonresponse	1	1.7

^a^Data available for 59 women

^b^Data available for 57 women

Self-reported measures were administered to incarcerated women to assess the severity of depression and anxiety symptoms over a two-week period and the severity of perceived stress over a four-week period. At the time of the survey, half of the women met the GAD-7 cut-off point for anxiety, presenting with moderate (20.0%) to severe (30.0%) levels of anxiety. Additionally, close to two thirds of women met the PHQ-9 cut-off point for depression; 20.0% presented with moderate depressive symptoms, 23.3% with moderately severe symptoms, and 20.0% with severe symptoms. Last, three quarters of women presented with moderate (58.3%) to high (16.7%) levels of perceived stress.

### Health-risk Behaviours

Health-risk behaviours are detailed in Table 3. Substance consumption was frequent in this sample of incarcerated women, with 78.3% of participants categorized as active cigarette smokers; 61.7% of these active smokers even reported an increase in tobacco use since incarceration. Nicotine dependence among women active smokers was high, with almost half of them presenting with moderate (40.0%) to high (8.3%) levels of dependence.

**Table 3:** *Incarcerated Women’s Self-Reported Health-Risk Behaviours Prior to and During Incarceration (n = 60)*

Variables	Study sample	
	*n* or else specified	% or else specified
Smoking status		
Non-smoker	9	15.0
Former smoker	3	5.0
Active smoker	47	78.3
Nonresponse	1	1.7
Felt tobacco use increased since incarceration (% yes) ^a^	29	61.7
Nicotine dependence (FTND) ^a^		
Low dependence	5	8.3
Low to moderate dependence	10	16.7
Moderate dependence	24	40.0
High dependence	5	8.3
Partial assessment	3	6.4
Alcohol use prior to incarceration (% yes) ^b^	38	64.4
Levels of risk for alcohol use problems prior to incarceration (AUDIT) ^c^		
Low risk	20	52.6
Drinking in excess of low risk guidelines	4	10.5
Harmful and hazardous drinking	6	15.8
Possible alcohol dependence	8	21.1
Alcohol use in prison (% yes) ^b^	4	6.8
Drug use prior to incarceration (% yes) ^b^	29	49.2
Levels of risk for drug use problems prior to incarceration (DAST-10) ^d^		
Low level	5	17.2
Moderate level	6	20.7
Substantial level	12	41.4
Severe level	6	20.7
Drug use in prison (% yes) ^b^	5	8.3
Prostitution experience prior to incarceration (% yes) ^e^	8	13.3
Sexual activity during incarceration (% yes) ^b^	7	11.9
Self-reported physical activity in prison ^b^		
More active than before incarceration	6	10
As active as before incarceration	6	10
Less active than before incarceration	46	76.7
Doesn’t know	1	1.7

^a^Percentages calculated on the number of active smokers, *n* = 47

^b^Data available for 589 women

^c^Percentages calculated on the number of women who drank prior to incarceration, *n* = 38

^d^Percentages calculated on the number of women who used drug prior to incarceration, *n* = 29

^f^Data available for 58 women

Thirty-eight women reported they drank alcohol during the 12 months prior to incarceration. According to their AUDIT score, 21.1% of these women were at risk for alcohol dependence, 15.8% had a harmful and hazardous consumption and 10.5% were drinking in excess of recommended guidelines. Since incarceration, only four women (6.8%) reported they had used alcohol, either drinking while on an unescorted prison furlough or drinking self-made “prison wine”.

Almost one in two (49.2%) women reported they used an illicit drug (i.e. heroin, cocaine, cannabis, or non-prescribed medication) during the 12 months prior to incarceration. According to their DAST-10 score, 20.7% of these women were at moderate risk for drug-related problems, 41.4% were at a substantial risk and 20.7% were at severe risk. Additionally, five women (8.3%) disclosed consumption of an illicit drug during incarceration (i.e. drugs or non-prescribed medication taken in prison or while on prison furlough). Of note, during the study period, no prison needle and syringe exchange programme was available to women incarcerated at this facility.

Thinking about sex-risk behaviours, eight women disclosed an experience with prostitution prior to incarceration. Self-report sexual behaviour in prison was particularly low in this sample, with 88.1% women indicating no sexual activity (i.e. sex or masturbation).

Last, women in *La Tuilière* usually have 3–4 periods of physical activity a week, yet 76.7% of the study sample reported a decrease in physical activity since incarceration. The reasons they gave were mainly lack of motivation (45.0%) or time (33.0%), whereas 5.0% of women explained their decrease in physical activity by the unavailability of the desired sports equipment (data not presented in tabular form). Only 10.0% of women declared to be more active than before their incarceration.

### Medication use and healthcare history

According to their medical chart, 85.0% of incarcerated women were taking medication on a regular basis (Table 4). Specifically, 50.0% of women were taking medication for a physical health problem and 81.7% for a mental health problem (i.e. benzodiazepine, antidepressants, neuroleptics), among which 15 women receiving opioid substitution therapy (i.e. methadone or Subutex®). Substitution medication is administered under medical surveillance, meaning that these 15 women have to swallow the pill in front of medical staff in the prison medical unit. Additionally, medical surveillance was also imposed to seven women who were to take their medication under surveillance due to cognitive problems, risks of traffic, or suicidal risk. Altogether, 46.7% of women were receiving medication for both physical and mental health problems at the time of the study. Of note, half of the medications for physical health conditions and 40.0% of those for mental health conditions were prescribed to women participants for the first time at *La Tuilière*, and a third of the opioid substitution therapies began in prison (data not presented in a tabular form).

**Table 4:** *Incarcerated Women’s Use of Medication and Healthcare History According to Medical Charts Data (n = 60)*

Variables	Study sample	
	*n*	%
*Use of medication*		
Use of medication (% yes)	51	85.0
For a physical health condition (% yes)	30	50.0
For a mental health condition incl. Substitution therapy (% yes)	49	81.7
For both physical and mental health conditions (% yes)	28	46.7
Medication administered under medical surveillance ^a^		
Substitution therapy (% yes)	15	29.4
Other medication (% yes)	7	13.7
*Healthcare needs history*		
Healthcare for a physical condition		
Only before incarceration (% yes)	2	3.3
Before and during incarceration (% yes)	31	51.7
Only during incarceration (% yes)	24	40.0
Healthcare for a psychiatric condition		
Only before incarceration (% yes)	2	3.3
Before and during incarceration (% yes)	20	33.3
Only during incarceration (% yes)	30	50.0

^a^Percentages calculated on the number of women who were taking medication, *n* = 51

**Fig. 1:**
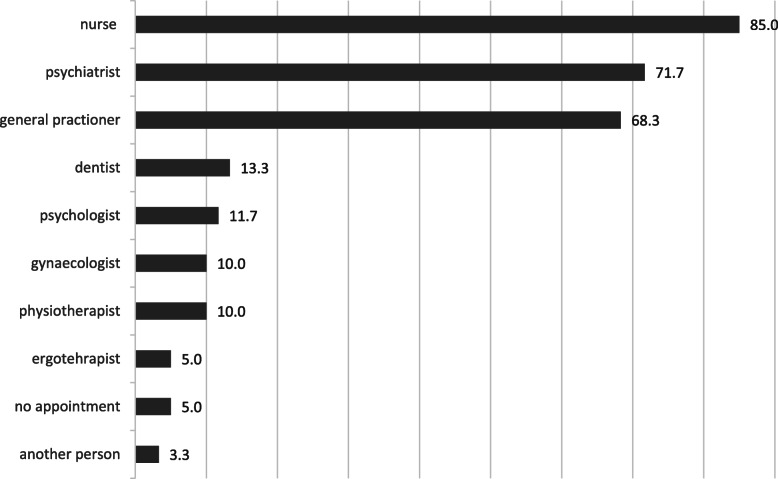
*Incarcerated Women’s Utilization of Prison Healthcare in the Previous 4 Weeks (n = 60)*

Healthcare utilization by incarcerated women over a four-week period prior to the survey is detailed in Fig. 1. Eighty-five percent of women reported at least one consultation with a prison nurse during those four weeks; this percentage comprises only one mandatory entry examination for the woman who was incarcerated during the four weeks preceding the survey (i.e., protocol violation). Further, 68.3% of women met at least once with a GP and 71.7% with a psychiatrist or a psychologist over a four-week period prior to the survey. Only three out of 60 women participants did not meet with any medical staff during the survey reference period.

## Discussion

To our knowledge, this study is among the first to comprehensively assess the health status and needs of women incarcerated in Switzerland using face-to-face survey interviews. As evidenced in North American and European literature (e.g., Antonetti et al., [2]; Binswanger et al., [5]; MacDonald, [40]; Plugge & Fitzpatrick, [51]), our findings demonstrate that incarcerated women in Switzerland also are a vulnerable population whose life trajectories are characterized by a low socio-economic status and overall poor health. Women in prison suffer from more complicated health problems than men, which critically calls for a gender-sensitive approach (Lewis, [37]; van den Bergh et al., [61]). Epidemiological knowledge obtained from general, non-institutionalized population cannot be transposed to the prison system due to the uniqueness of the female prison population (Binswanger et al., [5]).

Diagnoses of physical and mental comorbidities are frequent in prison research: our study was no exception, with 35.0% of women reporting both physical and mental health problems. Rheumatic diseases (15.0%), metabolic syndromes (13.3%), depressive (20.0%) and anxiety disorders (20.0%) were the most frequently reported health problems (data not presented in tabular form). Additionally, 16.7% of women were diagnosed with a somatic disorder while in prison (e.g., metabolic syndromes) and 40.0% were diagnosed with a new psychiatric disorder (e.g., adjustment and sleep disorders; data not presented in a tabular form). Further, anxiety, depression, and perceived stress scores were above clinical cut-off points for more than half of interviewed women. While not surprising, this report is concerning as mental health problems are among those factors that complicate reintegration. These mental health problems are often associated with women’s trauma histories (Grella et al., [22]; Moloney et al., [44]) and the uniqueness of women’s criminal trajectories (Nuytiens & Christiaens, [46]; Wright, Van Voorhis, Salisbury, & Bauman, [67]). Imprisonment often exacerbates mental health problems by failing to address the underlying trauma and particular mental health needs of women in prison (Moloney & Moller, [43]).

Not only were physical and mental health problems highly prevalent in this study, but women also shared numerous health-risk factors, which does not bode well for their future health. Behaviours such as smoking, alcohol and drug use problems, and low physical activity were frequent among women prior to their incarceration; smoking and sedentary lifestyle even worsened during incarceration. First, most women in this study were active smokers, specifically 78.3% compared to around 30.0% in the Swiss population (in the same age and sex category) (Kuendig, Notari, & Gmel, [36]). Yet women who are incarcerated, like men, spend most of their time in their cells, which are considered private spaces and therefore not subjected to smoking ban regulations in Switzerland. Non-smokers may share the cells of smokers and thus be highly exposed to second-hand smoke, which is associated with risks of cardiovascular disease, cancer, and pulmonary disease (Öberg, Jaakkola, Woodward, Peruga, & Prüss-Ustün, [47]). As a result, for example, smoking was recently banned in all Scottish prisons and preliminary results are encouraging showing an impact on the health of both incarcerated persons and staff (Semple, Dobson, Sweeting, Brown, & Hunt, [55]).

Second, while their declared alcohol and drug use in prison were low (6.8% and 8.3%, respectively), women in this study exhibited a high prevalence of alcohol and drug use problems, similar to the one reported in the literature. Over one in five women (21.1%) who reported they were drinking prior to their incarceration were at risk for alcohol dependence and 15.8% had a harmful and hazardous consumption; these numbers far exceed those found for incarcerated men (Moschetti et al., [45]) and in the general population (Gmel, Kuendig, Notari, & Gmel, [21]). During the same period, 49.2% women had used an illicit drug and almost two thirds of these women were at substantial risk (41.4%) or severe risk (20.7%) for drug use problems. Further, a third of women had been diagnosed with mental and behavioural disorders due to psychoactive substance use and three were diagnosed while in prison (data not presented in tabular form). Substance use problems may increase women’s early difficulties adjusting to prison, with possible withdrawal symptoms, aggressiveness, or comorbidity, thus complicating the provision of healthcare (Houser & Belenko, [27]). Furthermore, incarcerated women do not always have access to substance abuse treatment in prison and very few gender-responsive programs are available in correctional settings. Consequently, many women return to the community without having received substance abuse treatments (Zurhold & Haasen, [68]).

Third, at the time of their incarceration, almost a third of women participants were overweight or obese according to their BMI. And this study found an increase of the percentage of overweight women when comparing these initial BMI values to the ones collected at the time of the survey. A negative change that could be explained by a reduction of physical activity, diet modifications, and/or a prescription for neuroleptics, but also psychological distress.

### Access to health Services in Prison and Health Literacy

Many incarcerated women in this study undeniably experienced complicated health problems. The majority of them, however, had recently accessed prison medical services. More than 80.0% of women had met at least once with a psychiatrist or a psychologist since their incarceration, and 71.7% in the previous four weeks. More than 90.0% of women had met at least once with a GP, 68.3% in the four weeks prior to the study survey. It is also worth noting that the overall mean number of consultations was much higher for psychiatric care compared to somatic care (24.1 vs. 6.4 consultations) which suggests that independently of diagnosed mental health problems, women had a higher utilization of psychiatric healthcare. This can be partly explained by the fact that psychiatrists were present on site in prisons while GPs came by appointment only. That being said, although our data show that most women participants did meet at least once with a medical professional, our design does not allow us to answer whether women’s use of healthcare was indeed adequate to their needs. It would be interesting for future studies to provide a more nuanced examination of incarcerated women’s – and men’s – use of healthcare in Switzerland. Examining the reasons for possible gender differences in the type of health services both available and used (e.g., women’s greater psychiatric problems, healthcare access inequalities, gendered differences in terms of social desirability) is a first step towards ensuring we are addressing the specific needs of both women and men.

Use of medication in this sample was high, with almost half of incarcerated women having a prescription for both mental and physical health problems. Moreover, half of all treatments were introduced de novo during incarceration. These findings are likely associated with the frequency of depressive and anxiety symptoms and reported sleep difficulties. Yet neuroleptic and anxiolytic treatments are known to lead to dependence and metabolic diseases (Saravane et al., [54]). Alternate therapies (e.g., yoga, mindfulness) should be considered to help women mitigate their symptoms and improve their sleep, thus contributing to reducing drug prescriptions (Ferszt, Miller, Hickey, Maull, & Crisp, [19]).

### Strengths and limitations

Our findings are limited to the experiences of women in a single Swiss prison and thus may not necessarily apply to other prisons. We found a selection bias in our sample with significant differences in terms of age (i.e. a larger proportion of women in the 26–30 age group in our sample compared with the total female prison population) and in terms of nationality, the latter likely being the result of the lack of interpreters. As a consequence, women of Swiss origin were overrepresented (45.0%) in our study compared with the female inmate population at the research site, where 31.0% of inmates were from Swiss origin, 41.0% from European origin and 18.0% from an African origin. By excluding women with a more precarious situation (due to a migrant status or mental disorders) we might have overestimated the health of women inmates. Because of the small number of non-European women participants and the limited size of our sample, we were not able to investigate special cultural needs in prison or needs associated with the experience of migration. However, we believe that an intersectional approach would be of interest in prison health. Furthermore, given the limited sample, point estimates of prevalence are likely to be imprecise and statistical models may not be robust enough to assess determinants of health or access to healthcare. We used face-to-face interviews to administer our survey which potentially exacerbated stigmatization and social desirability bias in comparison with the use of written anonymous questionnaires. Our approach improved the quality of our data by ensuring the comprehension of survey questions and limiting nonresponse. However, it might have influenced women’s responses leading to them underreporting certain behaviours (e.g., substance use, trauma experiences) if they felt guilt or shame. Last, the cross-sectional design of the study does not allow for causal evidence of the effects of incarceration on the behaviour and health of incarcerated women.

Despite these limitations, our study is one of the few that addresses the physical and mental health of women incarcerated in a Switzerland. We used validated and comprehensive assessments to allow for comparisons with other prison populations and combined different data sources (i.e. medical charts and personal structured interviews) to better prevent reporting bias from both health professionals and incarcerated women.

## Conclusions

This study demonstrated incarcerated women’s poor health, health-risk behaviours, and overall low socio-economic outcomes. Structural changes in prisons, such as smoking bans or health promotion interventions to address obesity and stress could help improve the physical and mental health of incarcerated women. These are necessary but not sufficient. Access to healthcare resources in prison is also important and a supporting and non-stigmatizing and gender-responsive healthcare management is needed. Even if undesired, incarceration could represent an opportunity for women to have access to healthcare for their pre-existing medical conditions and to benefit from adequate medical and psychosocial care to prevent further health decline. The high prevalence of mental health problems calls for specific interventions, in particular those who integrate a focus on women’s trauma histories and correlates of psychological distress and risk-behaviours (Fournier, Hughes, Hurford, & Sainio, [20]). Particularly because health problems and substance abuse complicate reintegration (Kouyoumdjian et al., [32]); former incarcerated persons, both women and men with health problems are more likely to encounter obstacles in accessing employment, housing, and medical care when returning to the community (Mallik-Kane & Visher, [41]). Therefore, quality gender-responsive healthcare in prison is central to public health. Fighting low health literacy and increasing incarcerated women’s empowerment is key to improve their health, by helping them gain the capacity to understand basic health information to make appropriate health decisions when they return to the community (Berkman, Sheridan, Donahue, Halpern, & Crotty, [4]). Structured support and coordinated planning with community services is needed to prevent incarcerated women from returning to poor health and facilitate their transition into the community (Boutwell, Kendrick, & Rich, [6]).

## Data Availability

The datasets generated during the current study are not publicly available due to ethical restrictions. Indeed, participants’ name and personal information have been coded but a complete anonymization was not possible and the datasets contains private and sensible health data that cannot be made publicly available. These data are available from the corresponding author on reasonable request.

## Abbreviations

AUDIT: Alcohol use disorders test
BMI: body mass index
DAST-10: Drug Abuse Screening Test
DSM-IV: Diagnostic and Statistical Manual of Mental Disorders
FTND: Fagerström Test of Nicotine Dependence
GAD-7: General Anxiety Disorder
GP: general practitioner
IQR: interquartile range
M: mean
max: maximum
Mdn: median
min: minimum
PHQ-9: Patient Health Questionnaire
PSS-10: Perceived Stress Scale
SD: standard deviation
STIs: sexually transmitted infections
WHO: World Health Organization
UNODC: United Nations Office on Drugs and Crime
